# Acceptor-influenced and donor-tuned base-promoted glycosylation

**DOI:** 10.3762/bjoc.8.46

**Published:** 2012-03-20

**Authors:** Stephan Boettcher, Martin Matwiejuk, Joachim Thiem

**Affiliations:** 1 Department of Chemistry, Faculty of Science, University of Hamburg, Martin-Luther-King-Platz 6, 20146 Hamburg, Germany; 2 Glycom A/S, c/o DTU, Building 201, Anker Engelunds Vej 1, DK-2800 Kgs. Lyngby, Denmark

**Keywords:** glycosylation, oxyanion, reactivity, regioselectivity

## Abstract

Base-promoted glycosylation is a recently established stereoselective and regioselective approach for the assembly of di- and oligosaccharides by using partially protected acceptors and glycosyl halide donors. Initial studies were performed on partially methylated acceptor and donor moieties as a model system in order to analyze the key principles of oxyanion reactivities. In this work, extended studies on base-promoted glycosylation are presented by using benzyl protective groups in view of preparative applications. Emphases are placed on the influence of the acceptor anomeric configuration and donor reactivities.

## Introduction

For the assembly of oligosaccharides the complex and challenging control of regio- and stereochemistry has to be solved. Various contributions have facilitated enormously the access to complex oligosaccharide structures so far; however, regioselective and stereoselective glycosylations remain difficult and alternative concepts are welcome, and should be considered and studied.

Recently regioselective and β-stereospecific glycosylations employing saccharide oxyanions have been presented. Initial studies on base-promoted glycosylations were elaborated on partially methylated glucopyranosides as model systems. This systematic approach allowed determination of the preferred positions for glycosylation and the establishment of an oxyanion reactivity scale. In addition to studies with different base promoters and donors, the first successful conversions with benzylated acceptor and donor moieties were reported [1–3].

This novel glycosylation method could substantially reduce the need to apply protecting-group chemistry. Also, the β-specificity and base-promoted activation without any heavy-metal salt or Lewis acid are useful for synthetic applications. To develop the synthetic potential and evolve this method into an applicable alternative pathway towards di-, tri- or oligosaccharides, more research needs to be done.

So far, glycosylation reactions have been conducted by employing methyl α-glycopyranoside acceptors. Therefore, it was of particular interest to test the base-promoted glycosylation methodology with methyl β-glycopyranosides. Hence monobenzylated methyl α- and β-D-glycopyranosides were synthesized, then fucosylated by employing the base-promoted glycosylation methodology, and analysed with respect to the absolute and relative yields.

In addition, donor reactivity in base-promoted glycosylation reactions was investigated by employing partially benzylated methyl α- and β-D-glycopyranosides and donor halide mixtures.

## Results and Discussion

**Figure 1:**
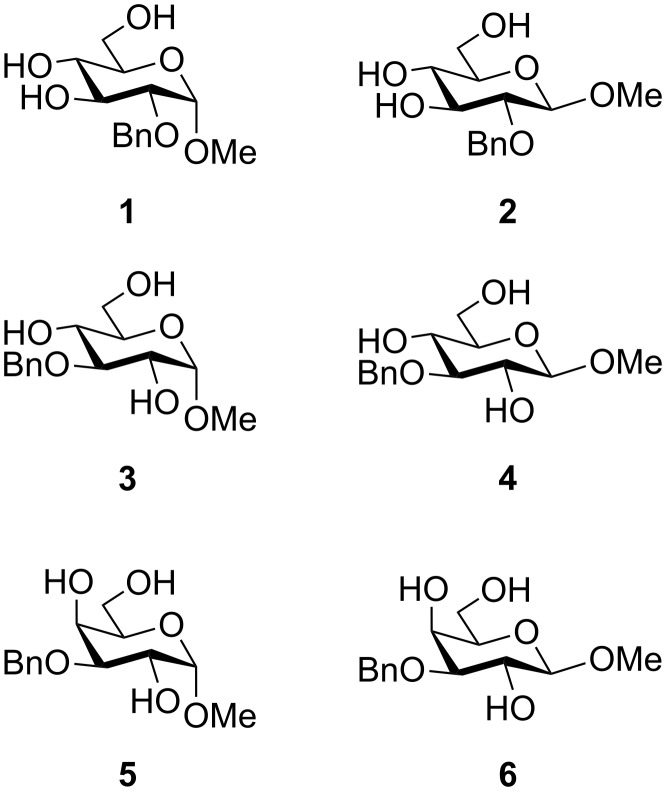
Monobenzylated methyl α- and β-D-gluco- and galactopyranoside acceptors **1**–**6**.

**Figure 2:**
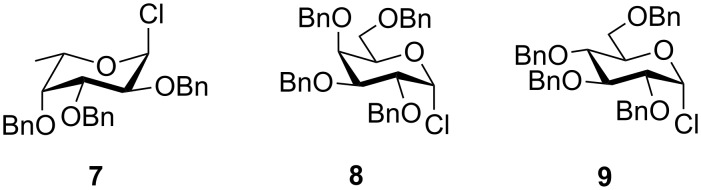
Benzylated glycopyranosyl halide donors **7**–**9**.

To answer the questions above, monobenzylated methyl α- and β-D-gluco- and galactopyranosides **1**–**6** were selected as acceptor moieties (Figure 1) and synthesized by employing standard protecting-group chemistry. After base activation of the acceptor hydroxy groups, glycosylation was performed with the perbenzylated glycopyranosyl chlorides **7**–**9** (Figure 2).

**Scheme 1:**
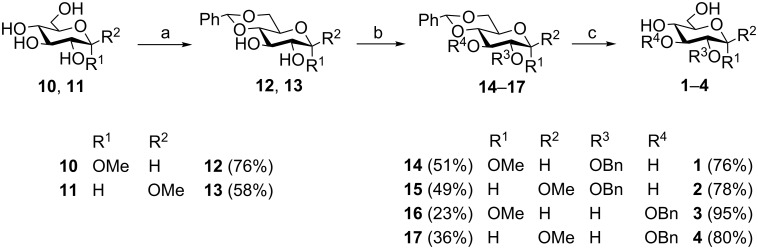
Synthesis of monobenzylated glucopyranosyl acceptors **1**–**4**. Reagents and conditions: (a) Benzaldehyde dimethylacetal, camphorsulfonic acid, ACN, 80 °C, 0.5–19 h; (b) NaOH, Bu_4_NHSO_4_, DCM, BnBr, 14–17 h, reflux; (c) MeOH, H_2_O, HCl, 2–22 h, reflux.

The synthesis of the monobenzylated glucopyranosyl acceptors **1**–**4** started with benzylidenation [4] of compound **10** and **11**, respectively (Scheme 1). As the next step, monobenzylation of **12** and **13** by phase-transfer catalysis [5] afforded derivatives **14**–**17**. Subsequent cleavage of the benzylidene protecting group [4] gave the target compounds **1**–**4** in yields of up to 95%.

**Scheme 2:**
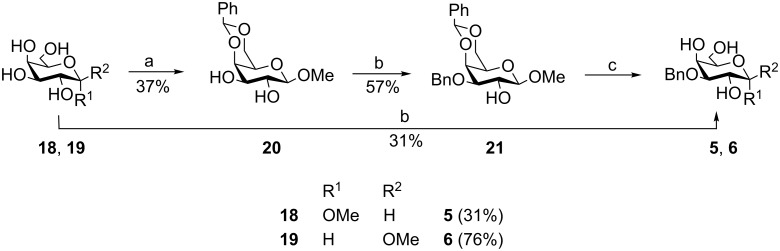
Synthesis of 3-*O*-monobenzylated gluco- und galactopyranosyl acceptors **5** and **6**. Reagents and conditions: (a) Benzaldehyde dimethylacetal, camphorsulfonic acid, ACN, 80 °C, 2.5 h; (b) dibutyltin oxide, toluene, BnBr, 24 h, reflux; (c) MeOH, H_2_O, HCl, 2–22 h, reflux.

Preparation of the 3-*O*-monobenzylated methyl α- and β-D-galactopyranosyl acceptors **5** and **6** was achieved via stannylidene acetals [6] (Scheme 2). Compound **18** was subjected to a one-step synthesis with Bu_2_SnO and BnBr, affording target compound **5**. The synthetic route for the methyl β-glycopyranoside acceptor **6** started with benzylidenation [4] of **19**, followed by monobenzylation via intermediate stannylidene acetal formation [6] and subsequent cleavage of the benzylidene group [4].

**Scheme 3:**
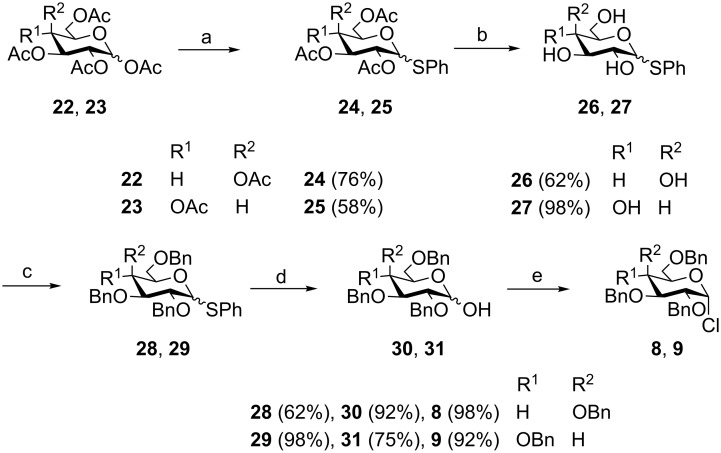
Synthesis of 2,3,4,6-tetra-*O*-benzyl-α-D-galactopyranosyl chloride (**8**) and 2,3,4,6-tetra-*O*-benzyl-α-D-glucopyranosyl chloride (**9**). Reagents and conditions: (a) 1. DCM, PhSH, BF_3_·Et_2_O, 0 °C, 1 h; 2. 4–5 d, rt; (b) MeOH, NaOMe, Amberlite IR 120 (H^+^), rt, 4–15 h; (c) 1. DMF, NaH, 0 °C, 1–2 h; 2. BnBr, 0 °C → rt, 16–40 h; (d) acetone/H_2_O (10:1), NBS, rt, −45 °C, 10 min to 5 h; (e) DCM, DMF, oxalyl chloride, 1 h, rt.

Formation of the perbenzylated α-fucopyranosyl chloride was achieved according to [2]. Starting with peracetylated derivatives **22** and **23**, a five-step synthesis was performed in each case to obtain the galacto- and glucopyranosyl donors **8** and **9** (Scheme 3). Initially, peracetates **22** and **23** were converted into the thioglycopyranosides **24** and **25** with BF_3_∙Et_2_O/thiophenole [7]. Subsequent deacetylation [8] and benzylation [9] afforded compounds **28** and **29**, whose thioglycosidic bonds were cleaved with NBS in water/acetone [10]. Finally, treatment with oxalyl chloride [11] gave the α-glycopyranosyl chlorides **8** and **9** in high yields.

The results of the base-promoted glycosylation of monobenzylated methyl α- and β-D-glycopyranosides with fucopyranosyl chloride **7** are summarized in Table 1. Fucosylation of the 2-*O*-benzylated α-derivative **1** gave predominantly the β-(1→6)-linked disaccharide **33**. Conversion of the 3-*O*-benzylated methyl α-D-glucopyranoside **3** resulted in β-(1→2) **34** and β-(1→6) disaccharides **35** in a ratio of ca. 1:9. By glycosylation of the 3-*O*-benzylated methyl α-D-galactopyranoside **5** with **7** only the β-(1→6)-linked disaccharide **37** was found. Thus, in all α-methyl glycopyranoside acceptors **1**, **3** and **5**, successful glycosidic bond formations with high regioselectivity towards position 6 were observed. These findings can be understood by the presence of adjacent hydroxy groups on acceptors **1**, **3** and **5**, such as 4,6-diol and/or 3,4,6-triol structures, respectively. After deprotonation the negative charge is dispersed by hydrogen bonding of unreacted hydroxy groups and located predominantly at position 6 due to the higher acidity of OH-6 [3]. Additionally, the basicity of the oxyanions stabilized by hydrogen bonding is decreased in comparison to isolated oxyanions, and this favours glycosidic bond formation over elimination.

**Table 1:** Glycosylation results for acceptors **1**–**6** with NaH as base and 2,3,4-tri-*O*-methyl-α-L-fucopyranosyl chloride (Fuc-Cl, **7**) as donor.^a^

Acceptor	Products	Yield [%]^b^	Relative yield [%]^c^			
			β-(1→2)	β-(1→3)	β-(1→4)	β-(1→6)

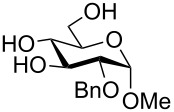 **1**	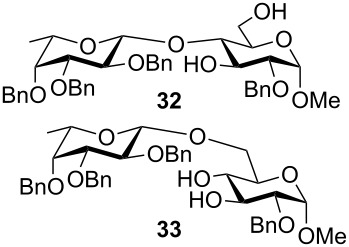	25	■^d^	–	40	60
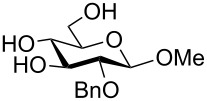 **2**	no reaction	–	■	–	–	–
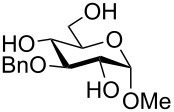 **3**	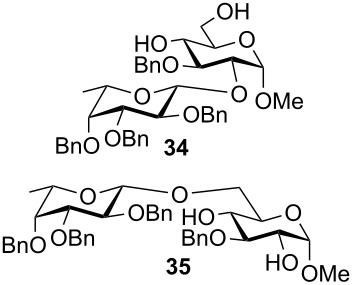	48^e^	13	■	–	87
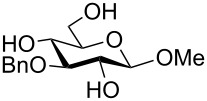 **4**	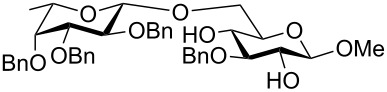 **36**	30	–	■	–	>98
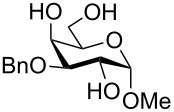 **5**	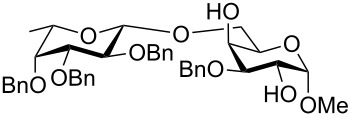 **37**	33	–	■	–	>98
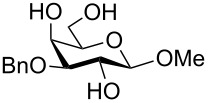 **6**	no reaction	–	–	■	–	–

^a^General reaction conditions: NaH (1.1–1.3 equiv), DMF, rt, 1 h, then donor **7** (1.0 equiv), rt, 4 h to 7 d; ^b^total yield of possible disaccharide regioisomers; ^c^after separation by column chromatography; ^d^■: benzylated position, no linkage possible; ^e^ [1].

Surprisingly glycosylation of the corresponding β-glucopyranosides **2**, **4**, and **6** only showed conversion for the 3-*O*-benzylated glucopyranosyl derivative **4**. Acceptors **2** and **6** did not react at all with the fucopyranosyl chloride **7**. Obviously, the anomeric configuration in the acceptor structure has a noticeable effect on the glycosylation, resulting in lower oxyanion reactivity for methyl β-glycopyranosides. The considerable effect of the anomeric configuration on the reactivity of remote hydroxy groups may be rationalized by stereoelectronic effects [12]. Moreover, the lower reactivities of the methyl β-glycopyranosides may also be dependent on the decreased solubility of the partially deprotonated methyl β-glycospyranosides in the solvent used.

The results of base-promoted glycosylations with donor mixtures are shown in Table 2. Reaction of the 3-*O*-benzylated α-D-glucopyranoside **3** with an equimolar mixture of the fuco- and galactopyranosyl donors **7** and **8** gave both β-(1→6)-linked disaccharides **35** and **38** in 20% yield, with a ratio of 85% in favour of the fucosylated product. The corresponding β-configured acceptor **4** yielded only the β-(1→6)-linked galactosylated disaccharide **39** in poor yield (5%). With the same acceptors, equimolar mixtures of the perbenzylated fuco- and glucopyranosyldonors **7** and **9** gave just the β-(1→6)-fucosylated products **35** and **36** in corresponding yields of 15% and 14%. Furthermore, a mixture of galacto- and glucopyranosyl donors **8** and **9** was employed, but this did not show any reaction at all. In all cases, disaccharide yields from donor mixtures were significantly lower; as rational of which, some unknown kind of interaction between the donors may be assumed.

**Table 2:** Reactivity studies of acceptors **3** and **4** with donors **7**–**9**.^a^

Acceptor	Donor (equimolar mixture)	Products	Yield [%]^b^	Ratio [%]

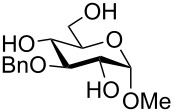 **3**	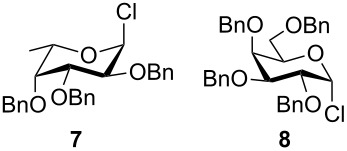	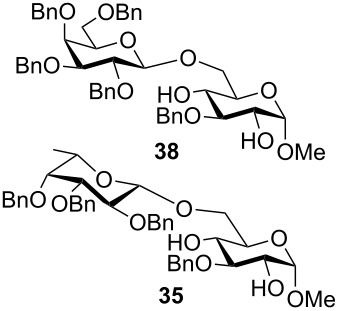	20	Gal/Fuc 15:85^c^
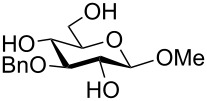 **4**	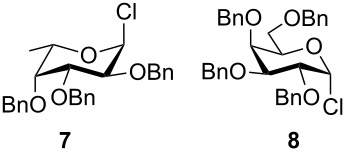	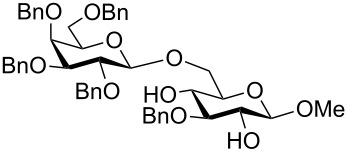 **39**	5	Gal only
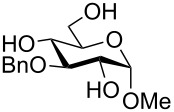 **3**	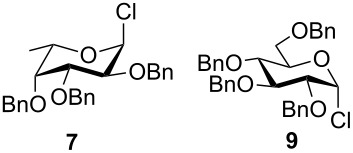	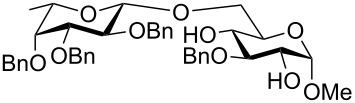 **35**	15	Fuc only
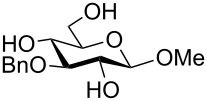 **4**	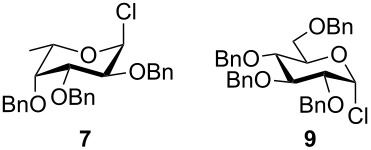	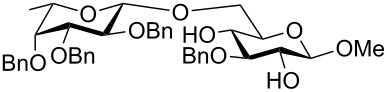 **36**	14	Fuc only

^a^General reaction conditions: NaH (1.1–1.3 equiv), DMF, rt, 1h, then donor **7** and **8** or **7** and **9** (1.0 equiv), rt, 4 h to 7 d; ^b^total yield of possible disaccharide regioisomers; ^c^ratio determined by ^1^H NMR.

## Conclusion

These studies gave evidence that the configuration of the anomeric centre of acceptors has a decisive influence on the base-promoted glycosylation approach, but which so far cannot be explained. Several α-monobenzyl acceptors (**1**, **3** and **5**) and their β counterparts (**2**, **4** and **6**) were synthesized and coupled with the perbenzylated fucopyranosyl donor **7**. Whereas all α-glycosidic acceptors gave disaccharides in moderate to acceptable yields, only the β-glycosidic acceptor **4** reacted, to give the dissacharide in much lower yield.

Furthermore, a comparison between the reactivities of several donor mixtures was established. For this purpose the perbenzylated donors **7**–**9** were synthesized and reacted in equimolar mixtures with acceptors **3** and **4**. The fucopyranosyl donor **7** showed the highest reactivity, followed by the galactopyranosyl donor **8**. The glucopyranosyl donor **9** showed no reaction at all. It turned out that the disaccharide yields from the mixtures were significantly lower, indicating unknown interactions between donors.

In cases with low yields or even no glycosylation products at all, elimination became the dominant reaction and led to the corresponding glycals. Hence, and based on the workup, the recovery of glycosyl halides was neither practicable nor achieved.

## Experimental

**General:** All reagents were purchased from commercial sources and used as received. Sodium hydride (NaH) was used as a 60% suspension in paraffin. TLC was performed on Merck silica gel 60 F_254_ plates. Compounds were detected by UV and/or by treatment with EtOH/H_2_SO_4_ (9:1) and subsequent heating. Column chromatography was performed with Merck/Fluka silica gel 60 (230–400 mesh). Solvents for column chromatography were distilled prior to use. ^1^H and ^13^C NMR spectra were recorded with Bruker AMX-400 or Bruker AV-400 spectrometers (400 MHz for ^1^H, 100 MHz for ^13^C) and calibrated by using the solvent residual peak. In CDCl_3_ TMS was used for calibration. Melting points were measured with an Apotec melting point apparatus. Optical rotations were obtained by using a Krüss Optronic P8000 polarimeter (589 nm, 25 °C). HRMS (ESI) were recorded with a Thermo Finnigan MAT 95XL mass spectrometer. MS (MALDI–TOF) were recorded with a Bruker Biflex II (positive reflection mode, matrix: 2,5-dihydroxybenzoic acid). Relative yields of disaccharide mixtures were determined by integration of the signals in the ^1^H NMR spectra of the anomeric or other well-separated protons. The abbreviation “dd~vt” stands for a double doublet that turns into a virtual triplet because of similar coupling constants. Physical and NMR data for **34** and **35** were published previously [1].

**General Procedure: Base-promoted glycosylations:** The acceptor molecule (1.0 mmol) was dissolved in dry dimethylformamide (2–4 mL), and sodium hydride (1.1–1.3 mmol for each hydroxy group; as a 60% suspension in paraffin) was added. After being stirred for one hour under argon at room temperature, the donor (1.0 mmol for each hydroxy group; donor-mixtures 0.5 mmol of each donor) dissolved in dry dimethylformamide (2–4 mL) was successively added to the reaction. After stirring for 4–100 h, methanol (2 mL) was added. The crude product was purified by flash column chromatography (petroleum ether/ethyl acetate, 2:1 v/v).

**Methyl 3-*O*-benzyl-β-D-glucopyranoside (4):** Prepared by hydrolysis of **17** [5] with hydrochloric acid [2]. Compound **17** (3.49 g, 9.37 mmol), MeOH (80 mL), H_2_O (8.0 mL), 2 N HCl (1.0 mL), 21.5 h, reflux. Yield: 80% (2.13 g, 7.49 mmol), colourless solid, mp 105 °C. 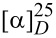 −7.7 (*c* 1.0, H_2_O); ^1^H NMR (400 MHz, MeOD) δ 7.47–7.43 (m, 2H, H_ar_), 7.36–7.24 (m, 3H, H_ar_), 4.92 (d, *J*_C(3)OC_*_H_*_2Ph-A,B_ = 11.1 Hz, 1H, C(3)-O-C*H*_2_-Ph-A), 4.88 (d, 1H, C(3)-O-C*H*_2_-Ph-B), 4.21 (d, *J*_1,2_ = 7.3 Hz, 1H, H-1), 3.90 (dd, *J*_6A,6B_ = 11.8 Hz, 1H, H-6A), 3.69 (dd, 1H, H-6B), 3.48–3.42 (m, 1H, H-3), 3.41–3.38 (m, 1H, H-4), 3.37–3.34 (m, 1H, H-2), 3.32–3.28 (m, *J*_5,6A_ = 2.3 Hz, *J*_5,6B_ = 5.8 Hz, 1H, H-5) ppm; ^13^C NMR (100 MHz, MeOD) δ 140.4, 129.2, 129.0, 128.5 (C_ar_), 105.5 (C-1), 86.3 (C-4), 77.9 (C-5), 76.0 (C(3)-O-*C*H_2_-Ph), 75.3 (C-2), 71.5 (C-3), 62.7 (C-6), 57.3 (-O-*C*H_3_) ppm; HRMS–ESI (*m*/*z*): [M + Na]^+^ calcd for C_14_H_20_O_6_Na, 307.1158; found, 307.1150.

**Methyl 2-*O*-benzyl-4-*O*-(2,3,4-tri-*O*-benzyl-β-L-fucopyranosyl)-α-D-glucopyranoside (32) and Methyl 2-*O*-benzyl-6-*O*-(2,3,4-tri-*O*-benzyl-β-L-fucopyranosyl)-α-D-glucopyranoside (33):** Prepared according to the general procedure. Compound **1** (70 mg, 0.25 mmol), NaH (31 mg, 0.77 mmol), **7** (335 mg, 0.739 mmol), DMF (20 mL). Yield: 43 mg (0.061 mmol, 25%), yellow sirup, relative yield **32**:**33** = 40:60 (^1^H NMR).

**32: **^1^H NMR (400 MHz, CDCl_3_) δ 7.31–7.19 (m, 20H, H_ar_), 4.89 (d, 1H, C(4’)-C*H*_2_-Ph-A), 4.80 (d, *J*_C(2’)OC_*_H_*_2Ph-A,B_ = 10.9 Hz, 1H, C(2’)-C*H*_2_-Ph-A), 4.77 (d, 1H, C(2’)-C*H*_2_-Ph-B), 4.70–4.53 (m, 5H, *J*_C(4’)OC_*_H_*_2Ph-A,B_ = 11.6 Hz, C(2)-C*H*_2_-Ph-A/B, C(3’)-C*H*_2_-Ph-A/B, C(4’)-C*H*_2_-Ph-B), 4.59 (d, *J*_1’,2’_ = 7.8 Hz, 1H, H-1’), 4.50 (d, *J*_1,2_ = 3.8 Hz, 1H, H-1), 4.02–3.96 (m, 1H, H-3), 3.96–3.90 (m, 1H, H-6A), 3.74 (dd~vt, 1H, H-2’), 3.62–3.56 (m, 1H, H-6B), 3.53–3.50 (m, 2H, H-4, H-5), 3.48–3.46 (m, 1H, H-4’), 3.46–3.40 (m, *J*_5’,6’_ = 6.7 Hz, 2H, H-3’, H-5’), 3.28 (dd, *J*_2,3_ = 9.6 Hz, 1H, H-2), 3.27 (s, 3H, -OC*H*_3_), 1.10 (d, 3H, H-6’) ppm; ^13^C NMR (100 MHz, CDCl_3_) δ 128.4, 128.4, 128.3, 128.2, 128.2, 128.1, 127.9, 127.8, 127.7, 127.7, 127.6 (C_ar_), 104.7 (C-1’), 98.4 (C-1), 82.4 (C-3’), 79.1 (C-2’), 78.9 (C-4), 78.5 (C-2), 76.2 (C-4’), 75.5 (C(2’)-O-*C*H_2_-Ph), 74.8 (C(4’)-O-*C*H_2_-Ph), 73.6 (C-3), 73.2 (C(3’)-O-*C*H_2_-Ph), 73.1 (C(2)-O-*C*H_2_-Ph), 70.9 (C-5’), 69.6 (C-5), 61.8 (C-6), 55.2 (-O*C*H_3_), 16.7 (C-6’) ppm.

**33: **^1^H NMR (400 MHz, CDCl_3_) δ 7.33–7.19 (m, 20H, H_ar_), 4.89 (d, 1H, C(4’)-C*H*_2_-Ph-A), 4.85 (d, 1H, C(2’)-C*H*_2_-Ph-A), 4.75–4.53 (m, *J*_C(2’)OC_*_H_*_2Ph-A,B_ = 10.9 Hz, *J*_C(4’)OC_*_H_*_2Ph-A,B_ = 11.6 Hz, 6H, C(2’)-C*H*_2_-Ph-B, C(2)-C*H*_2_-Ph-A/B, C(3’)-C*H*_2_-Ph-A/B, C(4’)-C*H*_2_-Ph-B), 4.55 (d, *J*_1,2_ = 3.8 Hz, 1H, H-1), 4.28 (d, *J*_1’,2’_ = 7.8 Hz, 1H, H-1’), 4.07–3.99 (m, 1H, H-6A/B), 3.88–3.85 (m, 1H, H-3), 3.76–3.70 (m, 1H, H-2’, H-5), 3.62–3.59 (m, 1H, H-4), 3.47–3.45 (m, 1H, H-4’), 3.44–3.39 (m, *J*_5’,6_ = 6.3 Hz, 2H, H-3’, H-5’), 3.27–3.24 (m, 1H, H-2), 3.23 (s, 1H, -OC*H*_3_), 1.10 (d, 1H, H-6’) ppm; ^13^C NMR (100 MHz, CDCl_3_) δ 138.8, 138.4, 137.8, 128.5, 128.5, 128.4, 128.2, 128.0, 127.9, 127.7, 127.6, 127.6, 127.5 (C_ar_), 104.2 (C-1’), 97.9 (C-1), 82.3 (C-3’), 79.4 (C-2’), 79.1 (C-2), 79.1 (C-5), 76.3 (C-4’), 75.0, 74.7, 73.2, 73.0 (-O-*C*H_2_-Ph), 72.6 (C-3), 70.6 (C-5’), 70.1 (C-4), 68.8 (C-6), 55.2 (-O*C*H_3_), 16.7 (C-6’) ppm; MALDI–TOF–MS (DHB, positive mode; *m*/*z*): [M + Na]^+^ calcd for C_41_H_48_O_10_Na, 723.3; found, 723.5.

**Methyl 3-*O*-benzyl-6-*O*-(2,3,4-tri-*O*-benzyl-β-L-fucopyranosyl)-β-D-glucopyranoside (36):** (a) Prepared according to the general procedure: Compound **4** (71 mg, 0.25 mmol), NaH (31 mg, 0.71 mmol), **7** (350 mg, 0.774 mmol), DMF (16 mL). Yield: 53 mg (0.074 mmol, 30%). (b) Prepared according to the general procedure by using a donor mixture: Compound **4** (65 mg, 0.23 mmol), NaH (30 mg, 0.75 mmol), **7** (150 mg, 0.330 mmol)/**9** (182 mg, 0.325 mmol), DMF (18 mL). Yield: 22 mg (0.031 mmol, 14%), yellow sirup. 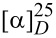 −106 (*c* 0.31, CHCl_3_); ^1^H NMR (400 MHz, CDCl_3_) δ 7.42–7.27 (m, 20H, H_ar_), 4.99 (d, *J*_C(4’)OC_*_H_*_2Ph-A,B_ = 11.6 Hz, 1H, C(4’)-O-C*H*_2_-Ph-A), 4.93 (d, *J*_C(2’)OC_*_H_*_2Ph-A,B_ = 10.7 Hz, 1H, C(2’)-O-C*H*_2_-Ph-A), 4.91 (d, *J*_C(3)OC_*_H_*_2Ph-A,B_ = 11.3 Hz, 1H, C(3)-O-C*H*_2_-Ph-A), 4.86 (d, 1H, C(3)-O-C*H*_2_-Ph-B), 4.79 (d, *J*_C(3’)OC_*_H_*_2Ph-A,B_ = 11.7 Hz, 1H, C(3’)-O-C*H*_2_-Ph-A), 4.76 (d, 1H, C(2’)-O-C*H*_2_-Ph-B), 4.72 (d, 1H, C(3’)-O-C*H*_2_-Ph-B), 4.69 (d, 1H, C(4’)-O-C*H*_2_-Ph-B), 4.36 (d, *J*_1’,2’_ = 7.5 Hz, 1H, H-1’), 4.19 (d, *J*_1,2_ = 7.5 Hz, 1H, H-1), 4.15 (dd, *J*_6A,6B_ = 11.0 Hz, 1H, H-6A), 3.86–3.79 (m, 3H, H-2’, H-4, H-6B), 3.57–3.38 (m, *J*_5,6A_ = 4.1 Hz, *J*_5’,6A_ = 6.6 Hz, 6H, H-2, H-3, H-3’, H-4’, H-5, H-5’), 3.51 (s, 1H, -OCH_3_), 1.17 (d, 3H, H-6’) ppm; ^13^C NMR (100 MHz, CDCl_3_) δ 138.7, 138.6, 138.5, 138.5, 138.4, 128.7, 128.5, 128.5, 128.4, 128.3, 128.2, 128.1, 128.0, 127.9, 127.9, 127.8, 127.6 (C_ar_), 104.0 (C-1’), 103.7 (C-1), 83.5 (C-3) 82.4 (C-3’), 79.0 (C-2’), 76.2 (C-4’), 75.2, 74.7, 74.6, 73.2 (-O-*C*H_2_-Ph), 74.6 (C-5), 73.9 (C-2), 70.8 (C-4), 70.6 (C-5’), 69.1 (C-6), 56.9 (-OCH_3_), 16.7 (-CH_3_) ppm; HRMS–ESI (*m*/*z*): [M + Na]^+^ calcd for C_41_H_48_O_10_Na, 723.3145; found, 723.3136.

**Methyl 3-*O*-benzyl-6-*O*-(2,3,4-tri-*O*-benzyl-β-L-fucopyranosyl)-β-D-galactopyranoside (37):** Prepared according to the general procedure. Compound **5** (72 mg, 0.25 mmol), NaH (26 mg, 0.65 mmol), **7** (343 mg, 0.731 mmol), DMF (10 mL). Yield: 58 mg (0.083 mmol, 33%), colourless solid, mp 98 °C. 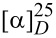 +72 (*c* 0.2, CHCl_3_); ^1^H NMR (400 MHz, CDCl_3_) δ 7.42–7.25 (m, 20H, H_ar_), 4.99 (d, 1H, -O-C*H*_2_-Ph), 4.90 (d, 1H, -O-C*H*_2_-Ph), 4.82–4.75 (m, *J*_1,2_ = 4.0 Hz, 3H, H-1, -O-C*H*_2_-Ph), 4.73 (d, 1H, -O-C*H*_2_-Ph), 4.71 (d, 1H, -O-C*H*_2_-Ph), 4.56 (s, 2H, -O-C*H*_2_-Ph), 4.40 (d, *J*_1’,2’_ = 7.8 Hz, 1H, H-1’), 4.16–4.13 (m, 1H, H-4), 4.01 (dd, *J*_6A,6B_ = 8.8 Hz, 1H, H-6A), 3.98 (dd, *J*_2,3_ = 9.8 Hz, 1H, H-2), 3.93–3.89 (m, *J*_5,6A_ = 4.3 Hz, *J*_5,6B_ = 8.6 Hz, 1H, H-5), 3.86 (dd, 1H, H-6B), 3.82 (dd, *J*_2’,3’_ = 9.5 Hz, 1H, H-2’), 3.62–3.55 (m, 2H, H-3, H-4’), 3.53 (dd, *J*_3’,4’_ = 3.0 Hz, 1H, H-3’), 3.51–3.44 (m, *J*_5’,6’_ = 6.5 Hz, 1H, H-5’), 3.39 (s, 3H, OCH_3_), 1.19 (d, 3H, H-6’) ppm; ^13^C NMR (100 MHz, CDCl_3_) δ 128.6, 128.5, 128.4, 128.3, 127.9, 127.8, 127.6, 127.6, 127.6 (C_ar_), 103.9 (C-1’), 99.5 (C-1), 82.5 (C-3’), 79.4 (C-2’), 78.4 (C-3), 76.2 (C-4’), 75.1, 74.6, 73.2, 71.7 (-O-*C*H_2_-Ph), 70.5 (C-5’), 68.6 (C-2), 68.1 (C-5), 67.3 (C-6), 66.0 (C-4), 55.4 (OCH_3_), 16.8 (C-6’) ppm; HRMS–ESI (*m*/*z*): [M + Na]^+^ calcd for C_41_H_48_O_10_Na, 723.3135; found, 723.3140.

**Methyl 3-*O*-benzyl-6-*O*-(2,3,4,6-tetra-*O*-benzyl-β-D-galactopyranosyl)-α-D-glucopyranoside (38):** (a) Prepared according to the general procedure: Compound **3** (70 mg, 0.25 mmol), NaH (32 mg, 0.80 mmol), **8** (415 mg, 0.742 mmol), DMF (20 mL). Yield: 20 mg (0.025 mmol, 10%). (b) Prepared according to the general procedure by using a donor mixture: Compound **3** (70 mg, 0.25 mmol), NaH (30 mg, 0.75 mmol), **7** (170 mg, 0.375 mmol)/**8** (208 mg, 0.372 mmol), DMF (20 mL). Yield: 34 mg (0.042 mmol, 20%). Relative yield **35**:**38** = 85:15. Slightly yellow sirup. 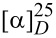 +53.4 (*c* 0.70, CHCl_3_); ^1^H NMR (400 MHz, CDCl_3_) δ 7.34–7.19 (m, 25H, H_ar_), 4.89–4.84 (m, 3H, -O-C*H*_2_-Ph), 4.73–4.61 (m, 5H, H-1, -O-C*H*_2_-Ph), 4.52 (d, 1H, -O-C*H*_2_-Ph), 4.41–4.31 (m, *J*_1’,2’_ = 7.5 Hz, 3H, H-1’, -O-C*H*_2_-Ph), 4.09–4.01 (m, 1H, H-6A), 3.83–3.80 (m, 1H, H-4), 3.77 (dd~vt, 1H, H-2’), 3.73–3.66 (m, 2H, H-5’, H-6B), 3.56–3.42 (m, 7H, H-2, H-3, H-3’, H-4’, H-5, H-6’A/B), 3.29 (s, 1H, -OC*H*_3_) ppm; ^13^C NMR (100 MHz, CDCl_3_) δ 138.7, 138.7, 128.5, 128.4, 128.4, 128.3, 128.2, 128.1, 128.1, 128.0, 127.9, 127.8, 127.6, 127.5, 127.5 (C_ar_), 104.2 (C-1’), 99.3 (C-1), 82.7 (C-3), 82.3 (C-3’), 79.3 (C-2’), 75.2, 74.9, 74.5, 73.6, 73.0 (-O-*C*H_2_-Ph), 73.5 (C-4), 73.5 (C-4’), 72.6 (C-2), 70.8 (C-5), 70.2 (C-5’), 69.2 (C-6), 68.7 (C-6’), 55.3 (-O*C*H_3_) ppm; HRMS–ESI (*m*/*z*): [M + Na]^+^ calcd for C_48_H_54_O_11_Na, 829.3564; found, 829.3545.

**Methyl 3-*O*-benzyl-6-*O*-(2,3,4,6-tetra-*O*-benzyl-β-D-galactopyranosyl)-β-D-glucopyranoside (39):** Prepared according to the general procedure by using a donor mixture: Compound **4** (65 mg, 0.23 mmol), NaH (29 mg, 0.73 mmol), **7** (151 mg, 0.334 mmol)/**8** (175 mg, 0.313 mmol), DMF (16 mL). Yield: 8.6 mg (0.011 mmol, 5%). Yellow sirup. 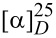 −9.6 (*c* 0.40, CHCl_3_); ^1^H NMR (400 MHz, CDCl_3_) δ 7.31–7.19 (m, 25H, H_ar_), 4.88–4.82 (m, 3H, -O-C*H*_2_-Ph), 4.74–4.69 (m, 2H, -O-C*H*_2_-Ph), 4.66–4.62 (m, 2H, -O-C*H*_2_-Ph), 4.52 (d, 1H, -O-C*H*_2_-Ph), 4.42–4.30 (m, 2H, -O-C*H*_2_-Ph), 4.37 (d, *J*_1,2_ = 3.5 Hz, 1H, H-1), 4.08–4.03 (m, *J*_6A,6B_ = 10.8 Hz, 1H, H-6A), 4.06 (d, *J*_1’,2’_ = 7.6 Hz, 1H, H-1’), 3.82–3.80 (m, 1H, H-4), 3.78–3.74 (m, 1H, H-2), 3.74 (dd, 1H, H-6B), 3.56–3.42 (m, *J*_5,6B_ = 6.0 Hz, 6H, H-3, H-4’, H-5, H-5’, H-6’A/B), 3.39 (dd, *J*_2’,3’_ = 9.8 Hz, 1H, H-2’), 3.36 (s, 1H, -OC*H*_3_), 3.31 (dd~vt, 1H, H-3’) ppm; ^13^C NMR (100 MHz, CDCl_3_) δ 138.6, 128.6, 128.4, 128.4, 128.3, 128.3, 128.2, 128.2, 128.2, 128.1, 128.0, 127.9, 127.8, 127.8, 127.6, 127.5 (C_ar_), 104.0 (C-1), 103.6 (C-1’), 83.7 (C-3’), 82.2 (C-3), 79.1 (C-2), 75.2, 74.7, 74.5, 73.5, 72.9 (-O-*C*H_2_-Ph), 73.5 (C-4), 74.6 (C-5), 74.2 (C-2’), 73.4 (C-4), 71.4 (C-5’), 69.3 (C-6), 68.7 (C-6’), 57.1 (-O*C*H_3_) ppm; MALDI–TOF–MS (DHB, positive mode; *m*/*z*): [M + Na]^+^ calcd for C_48_H_54_O_11_Na, 829.36; found, 830.1.
